# Room-Temperature Fabrication of a Nickel-Functionalized Copper Metal–Organic Framework (Ni@Cu-MOF) as a New Pseudocapacitive Material for Asymmetric Supercapacitors

**DOI:** 10.3390/polym11050821

**Published:** 2019-05-07

**Authors:** Yi Wang, Shengqiang Nie, Yuan Liu, Wei Yan, Shaomin Lin, Gang Cheng, Huan Yang, Jun Luo

**Affiliations:** 1 College of Chemistry and Material Engineering, Gui Yang University, Guiyang 550005, China; wy742011@hotmail.com (Y.W.); nieshq1987@163.com (S.N.); wangy49@mail2.sysu.edu.cn (Y.L.); lrasyw@163.com (W.Y.); tzchenggang2005@126.com (G.C.); 2 School of Material Science and Engineering, Han Shan Normal University, Chaozhou 521041, China; lsm@hstc.edu.cn

**Keywords:** nickel-functionalized metal–organic frameworks, asymmetric supercapacitor, energy storage

## Abstract

A nickel-functionalized copper metal–organic framework (Ni@Cu-MOF) was prepared by a facile volatilization method and a post-modification synthesis method at room temperature. The obtained Ni@Cu-MOF electrode delivered a high capacitance of 526 F/g at 1 A/g and had a long-term cycling stability (80% retention after 1200 cycles at 1 A/g) in a 6 M KOH aqueous solution. Furthermore, an asymmetric supercapacitor device was assembled from this Ni@Cu-MOF and activated carbon electrodes. The fabricated supercapacitor delivered a high capacitance of 48.7 F/g at 1 A/g and a high energy density of 17.3 Wh/kg at a power density of 798.5 kW/kg. This study indicates that the Ni@Cu-MOF has great potential for supercapacitor applications.

## 1. Introduction

With global energy demands and the high consumption of fossil fuels, accompanied by increasing environmental pollution, sustainable and renewable energy supplies are in high demand [1,2,3]. Supercapacitors (SCs) are energy storage devices (similar to lithium-ion batteries and fuel cells) that provide efficient renewable systems [4,5,6,7]. SCs have attracted widespread attention due to their large power density, fast charging and discharging time, and long life span [8,9,10].

According to the charge storage mechanism, SCs are divided into two types: electrical double-layer capacitors (EDLCs) and faradaic pseudocapacitors. In an EDLC, the ions are stored at the Helmholtz double-layer interface found on materials such as carbon nanotubes and carbon fibers [11,12,13]. In pseudocapacitors, Faradic redox reactions occur at the surface or near-surface of electroactive sites on, for example, transition metal oxides and hydroxides [14,15,16]. To achieve a higher capacitance, it is important to search for and design porous materials with better energy storage properties for SCs.

Metal–organic frameworks (MOFs) are composed of metal ions and organic ligands. They are porous materials with a remarkable surface area, permanent porosity, and metal cations that participate in various redox reaction. MOFs are used as promising electrode materials in SCs [17,18,19]. According to the energy storage required for the application, there are two types of SCs formed from MOFs: (1) MOFs used as controlled templates or precursors for synthesizing mesoporous carbon, metal oxides, and metal sulfide via thermolysis or sulfurization [20,21,22,23,24,25,26], and (2) MOFs used as electroactive materials for SCs owing to their porous properties. To improve the electrochemical performance of MOFs, the supercapacitive properties of MOFs should be further evaluated [27,28,29,30].

In recent years, bimetallic transition metal–organic frameworks with two kinds of metal cations have been considered as promising materials for supercapacitors [31,32,33] and electrocatalysts [34,35,36,37]. It has been proven that bimetallic MOFs exhibit a better electrochemical performance than monometallic MOFs, including an improvement in framework stability and in magnetic properties, which can be ascribed to the synergies or the enhanced charge transfer between different ions. Bimetallic MOFs could be utilized as ideal templates for the construction of bimetallic sulfides and metallic (or metal) oxides and are often accompanied by porous carbon, which is produced by the decomposition of linkers in the MOFs. Various inexpensive binary compounds composed of transition metal species have been developed as electrode materials with higher theoretical specific capacitances or capacities [38,39,40,41]. Thus, it is important to fabricate porous bimetallic MOFs with enhanced electrochemical performance, but it is also a great challenge [42,43]. The postsynthetic modification method (PSM) has been used to achieve the chemical modification of many organic and inorganic materials. As the PSM avoids the decomposition of structures and other side reactions, it has become a relatively mature method for introducing ions into an MOF. However, there have been no reports on the engineering of Nickel-functionalized copper metal–organic frameworks and their electrochemical performance.

In this study, a nickel-functionalized copper MOF (Ni@Cu-MOF) was fabricated through a facile volatilization method and a PSM. The as-fabricated Ni@Cu-MOF was used as the electrode material for SCs and exhibited a high specific capacity of 526 F g^−1^ at 1 A g^−1^, compared with Cu–MOF (126 F g^−1^ at 1 A g^−1^), owing to the synergy effect, great rate performance (428 F g^−1^ at 8 A g^−1^), and high cycle stability (80% retention over 1200 cycles at 1 A/g). In addition, a flexible asymmetric SC was built from the Ni@Cu-MOF and active carbon, and the specific capacity was 48.7 F/g at 1 A/g. The energy density of the asymmetric supercapacitor (ASC) was 17.3 W h kg^−1^, and the power density was 798.5 W kg^−1^.

## 2. Materials and Method

### 2.1. Synthesis of the Cu-MOF

A mixture of Cu(NO_3_)_2_ (0.05 mmol) and 2,6-pyridinedicarboxylic acid (0.05 mmol) in 10 mL deionized water was put into a container for 48 hours at room temperature; blue block crystals filled the entire liquid. The crystals were picked from the mother liquor, washed with deionized water (8 mL) three times, and dried in air.

### 2.2. Synthesis of the Ni@Cu-MOF

The powder of the Cu-MOF was immersed in an aqueous solution of 1 × 10^−3^ M Ni(NO_3_)_2_. After two days, the product (Ni@Cu-MOF) was obtained and washed with deionized water, then dried in air. The content ratio of Ni/Cu was 1:4.

### 2.3. Electrochemical Measurements

Electrochemical experiments including cyclic voltammetry (CV), galvanostatic charge−discharge tests (GCD), and electrochemical impedance spectroscopy (EIS) were conducted using a three-electrode system in a 6 M KOH solution. EIS experiments were recorded in the range of 0.01–10^5^ Hz. These experiments were carried out using a CHI660E electrochemical workstation.

### 2.4. Materials Characterization

The metal ion (Cu/Ni) content in the Cu@Ni-MOF was recorded by inductively coupled plasma mass spectrometry (ICP–MS) (Icap Qc). The electrochemical measurements were carried out on a CHI 660E potentiostat electrochemical workstation (Thermo-Fisher, Berlin, Germany). X-ray diffraction (XRD) patterns were recorded with a Rigaku Miniflex 600 X-ray diffractometer (Rigaku, Tokyo, Japan) from 5° to 50°. An infrared spectrum was recorded using an IR Affinity-1 FT-IR spectrometer (Shimadzu, Kyoto, Japan) in the range of 400–4000 cm^−1^. Cu(NO_3_)_2_, Ni(NO_3_)_2_, 2,6-pyridinedicarboxylic acid were bought commercially(Jinan Henghua Technology Co., Ltd., Jinan, China), acetylene carbon black and PolyVinylidene Fluoride were bought commercially(TIMCAL, Changzhou, China)

## 3. Results and Discussion

**Figure 1:**
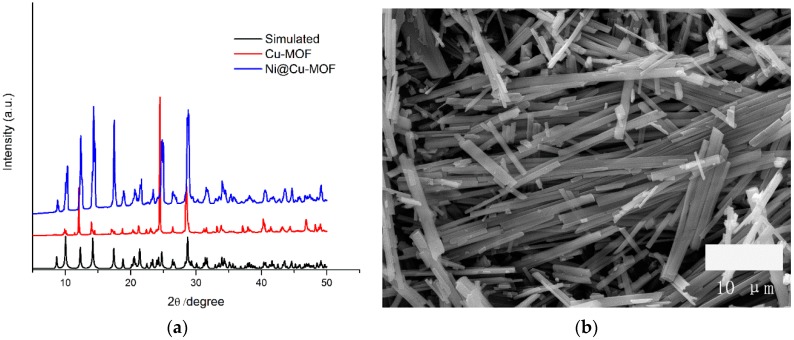

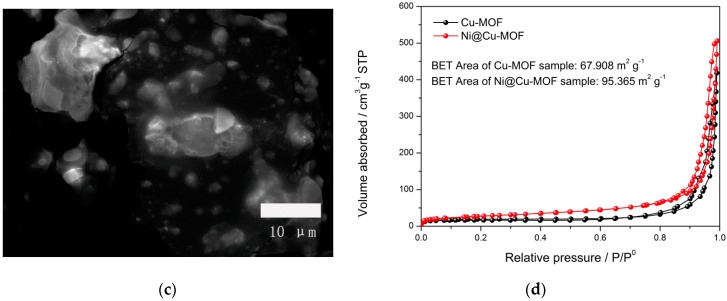
(**a**) X-ray diffraction (XRD) patterns of the simulated, as-prepared copper metal–organic framework (Cu MOF), and nickel-functionalized copper metal–organic framework (Ni@Cu-MOF), (**b**) SEM of the Cu MOF, (**c**) SEM of the Ni@Cu MOF, and (**d**) Nitrogen adsorption of the Cu-MOF and Ni@Cu-MOF BET.

Figure 1 shows the X-ray diffraction (XRD) patterns of the as-prepared Cu-MOF, Ni@Cu-MOF, and simulated Cu-MOF. The measured XRD patterns of the as-prepared Cu-MOF were in good agreement with the XRD pattern of the simulated Cu-MOF [44]. After introducing Ni^2+^ into the Cu-MOF, the PXRD pattern of the Ni@Cu-MOF remained unchanged. The results suggested that the Cu-MOF and the Ni@Cu-MOF were synthesized successfully. The SEM (Figure 1b) and TEM (Figure S1) images of the Cu-MOF revealed that the size of the Cu-MOF nanorod was about 230 nm. On the basis of the SEM (Figure 1c) and TEM (Figure S2) images of the Ni@ Cu-MOF after the introduction of Ni^2+^ into the Cu-MOF, we speculated that the Cu-MOF nanorod dissolved in the nickel nitrate aqueous solution; meanwhile, the phase structures of the Cu-MOF particles changed from nanorods to smaller nanoparticles.

The surface areas of the Cu-MOF (67.908 m^2^·g^−1^) and the Ni@Cu-MOF (95.365 m^2^·g^−1^) were calculated by a multipoint BET model. The increase in surface area of the Ni@Cu-MOF was attributed to the smaller size of the Ni@Cu-MOF. In the range of P/P_0_ = 0.8–1.0, the hysteresis loop in the isotherms showed that mesopores and macropores existed in the MOF. Obviously, the pore distribution curve suggested that the Ni@Cu-MOF also possessed mesopores and macropores, and the pore diameters were about 60–90 nm. In addition, these mesopores and macropores were helpful to the rapid diffusion of ions and promoted a rapid faradaic reaction and high rate performance.

**Figure 2:**
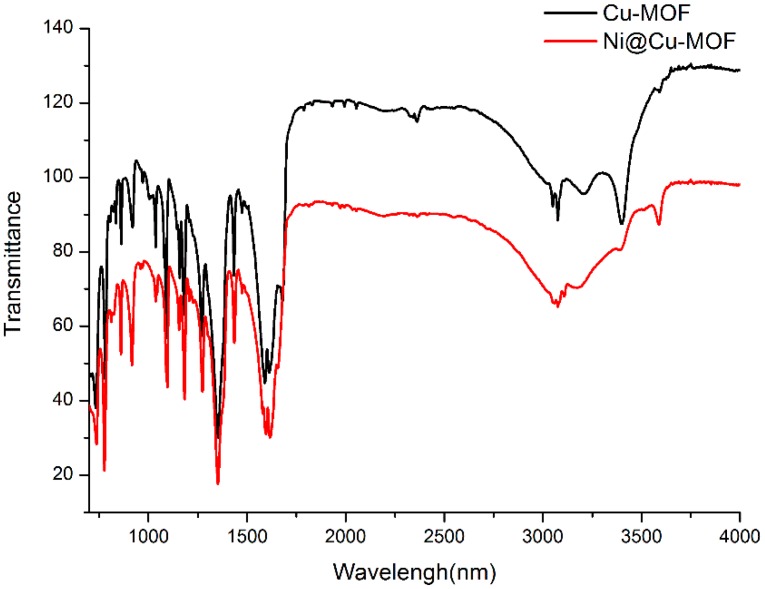
IR spectra of the Cu-MOF and the Ni@Cu-MOF.

Figure 2 shows the FT-IR spectra. Several bands between 3200 and 3500 cm^−1^ were characteristic of the n(O–H) mode of carboxyl groups. The absorption band at 1620 cm^−1^ belonged to the n(C=O) mode of carboxyl groups. In addition, the bands at 1300 and 920 cm^−1^ were assigned to the d(O–H) mode of carboxyl groups. The band at 1420 cm^−1^ belonged to the n(C=N) mode of carboxyl groups. After the introduction of Ni^2+^ into the Cu-MOF, the IR bands of the Ni@Cu-MOF were consistent with those of the Cu-MOF. The results showed that the introduction of Ni^2+^ did not influence the crystalline structure, as shown by the PXRD patterns.

**Figure 3:**
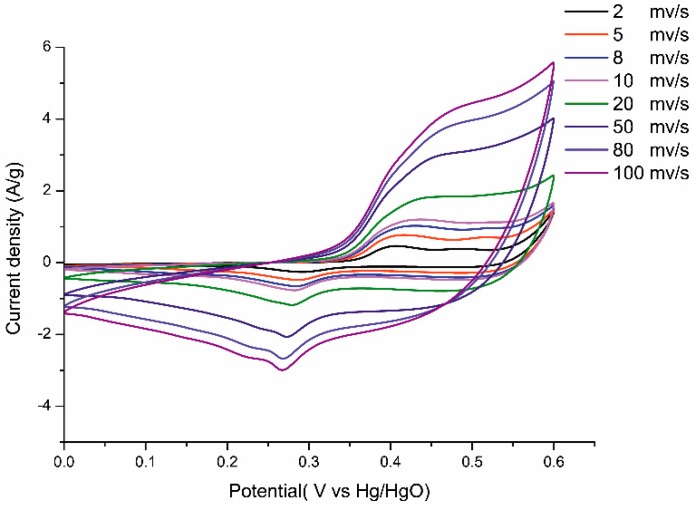
Cyclic voltammetry (CV) curves of the Ni@Cu-MOF at scan rates 2, 5, 8, 10, 20, 50, 80, and 100 mV s^−1.^

The energy storage capacitance of the as-prepared Ni@Cu-MOF was assessed by cyclic voltammetry (CV) measurements. Figure 3 shows the CV responses of the Ni@Cu-MOF at different scan rates (2–100 mV s^−1^). The CV curves of the Ni@Cu-MOF showed an integrated pair of redox peaks due to the redox reaction (Ni@Cu-MOF) in the 6 M KOH electrolyte solution (reversible valence-state changes between Ni^2+^ and Ni^3+^), which occurred due to the adsorption/desorption of OH^−^ in the layer during the electrochemical reaction. Furthermore, the peak current of the CV increased (scan rate from 2 to 100 mV s^−1^); accordingly, the current response increased. The results showed a good rate and reversibility of the fast charge–discharge response [17,45].

**Figure 4:**
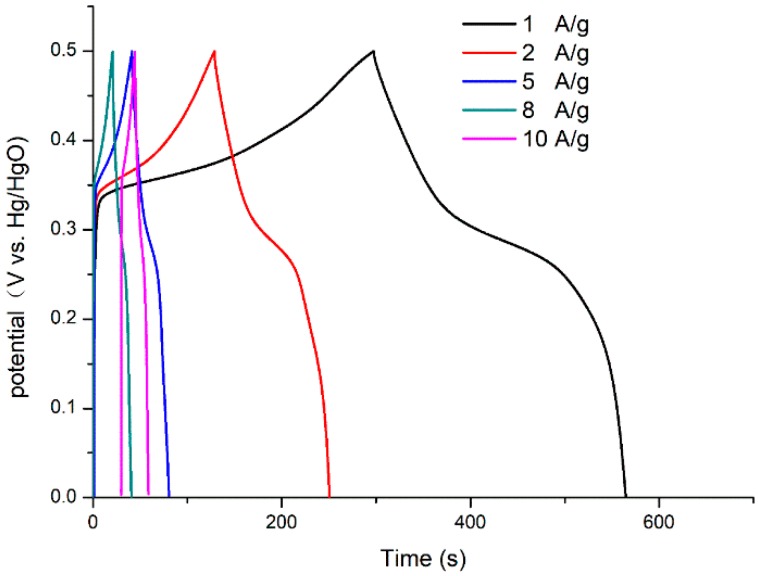
Galvanostatic charge–discharge curves of the Ni@Cu-MOF at different current densities.

Figure 4 shows the galvanostatic charge–discharge (GCD) curves of Ni@Cu-MOF and Cu-MOF at different current densities (ranging from 0 to 0.5 V). Specific capacitances of 526, 484, 390, 320, and 280 F g^−1^ were observed at 1.0, 2.0, 5.0, 8.0, and 10 A g^−1^, respectively. The discharge curves of the Ni@Cu-MOF and the Cu-MOF at 1 A g^−1^ were different from straight and flat curves, which indicated that the process of GCD can be ascribed to the Faradaic process. The GCD curves of Ni@Cu-MOF showed a better performance than the GCD curves of the Cu-MOF, reducing the polarization. As calculated, the specific capacitance of the Ni@Cu-MOF (526 F/g) was more than four times higher than the specific capacitance of the Cu-MOF (126 F/g) at 1 A/g (Figures S5 and S6).

**Figure 5:**
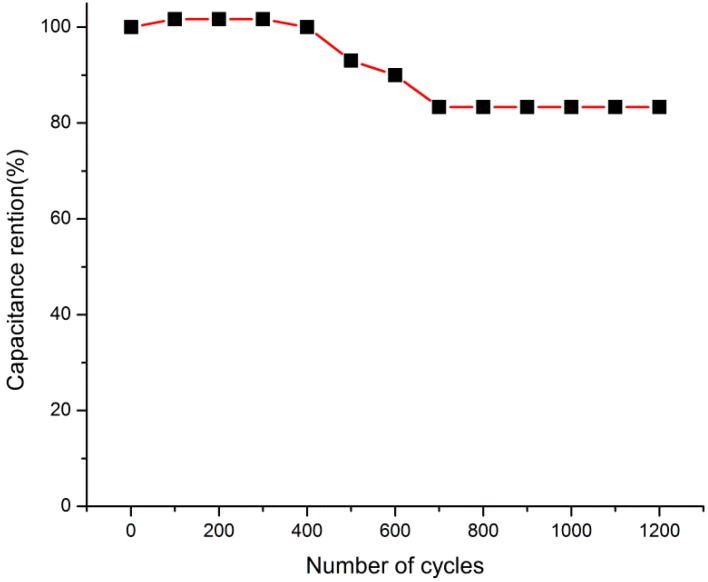
Cycle stability of the Ni@Cu-MOF-based supercapacitor at 1 A g^−1.^

The cycling tests of the Ni@Cu-MOF were also recorded by GCD measurements. Figure 5 shows the GCD curves of the Ni@Cu-MOF electrode at 1 A/g in a 6 mol/L KOH solution for 1200 cycles. The results showed that the Ni@Cu-MOF electrode maintained 80% of its specific capacitance after 1200 cycles, which demonstrated good long-term cycling stability and capacitance.

**Figure 6:**
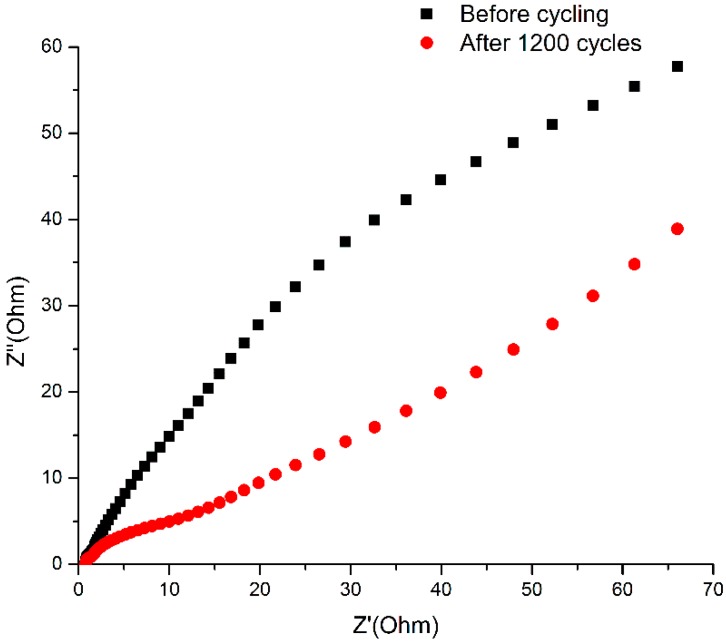
Nyquist plots of the Ni@Cu-MOF before and after 1200 cycles. Electrochemical properties of the Ni@Cu-MOF/activated carbon (AC) asymmetric supercapacitor.

Electrochemical impedance spectroscopy (EIS) (potential of 0.3046 V in a 6 M KOH solution) was studied in the frequency range of 0.01 Hz to 100 kHz. In Figure 6, the Nyquist plots of the Ni@Cu-MOF show a semicircle and linear curve in the high- and low-frequency regions, respectively. The semicircle represents Faradaic charge transfer processes (Rct). The internal resistance (Rs) was deduced from the real axis intercept (Z’) and included the intrinsic resistance (Ni@Cu-MOF), the bulk resistance of the solution, and the resistance of the Ni@Cu-MOF and Ni foam (the current collector). The Rct of the Ni@Cu-MOF was 0.75 Ω. After 1200 cycles, the value of the Rct (Ni@Cu-MOF) showed a slight increase from 0.75 to 0.77. The phase angle of the line (Ni@Cu-MOF after 1200 cycles) was similar to 45 °C in the low-frequency region, which resulted from the synergistic effect of the Ni@Cu-MOF.

**Figure 7:**
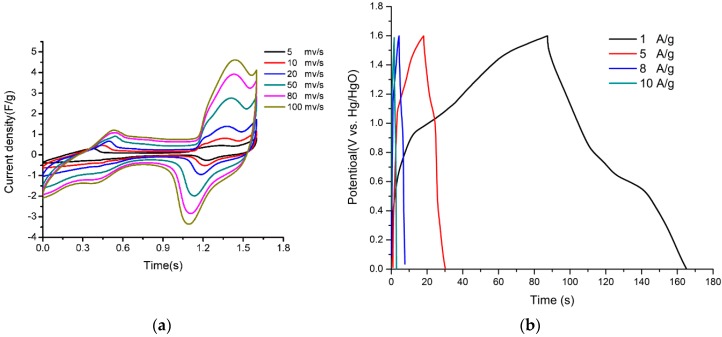
CV curves (**a**) and the charge–discharge curves (**b**) of the Ni@Cu-MOF/activated carbon asymmetric supercapacitor measured in a 6 mol L^−1^ KOH electrolyte solution.

To further explore the energy storage application of the Ni@Cu-MOF electrode, an ASC device was built with a positive electrode (Ni@Cu-MOF) and a negative electrode (activated carbon, AC). According to the CV results, in the three-electrode system in the 6 M KOH solution, the potential window of the Ni@Cu-MOF and AC was in the ranges of 0–0.6 V and −1.0 to 0 V (Figures S7 and S8). As shown in Figure 7a,b, the CVs at various scan rates from 2 to 100 mV/s and the GCD curves at various current densities from 1 to 1 0 A/g displayed a potential window from 0 to 1.6 V. The CV curve did not change at a high scan rate (100 mV s^−1^). The results showed that the ASC device has the potential for a high power delivery [24]. The specific capacitance (C), power density (P), and energy density (E) of the ASCs were calculated from the GCD curves at 1 A/g. As shown in Figure 7b, the ASCs achieved a potential as high as 1.6 V. Moreover, the value of C_s_ was calculated from the GCD curves, and a specific capacitance of 48.7 F/g at a 1 A g^−1^ was observed. The ASCs delivered a high energy density of 17.3 Wh/kg at a power density of 798.5 kW/kg.

**Figure 8:**
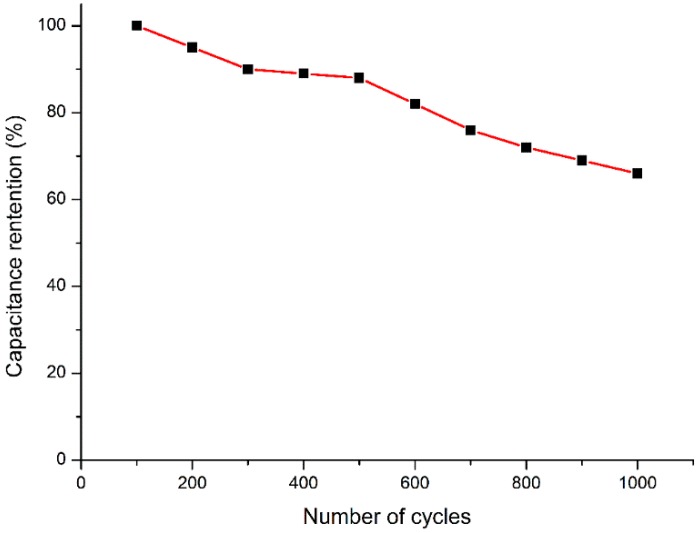
Cycle performance of the Ni@Cu-MOF-based asymmetric supercapacitor at a current density of 1 A/g.

Furthermore, cycling stability tests of the ASCs were evaluated at 1 A/g, as shown in Figure 8. After 1000 cycles, the capacitance of the ASCs was maintained at ~63%. Therefore, the Ni@Cu-MOF has potential for practical applications.

## 4. Conclusions

In summary, a hybrid bimetallic Ni@Cu-MOF was prepared by a facile volatilization method and a post-modification synthesis method at room temperature. The bimetallic Ni@Cu-MOF delivered a higher specific capacitance of 526 F g^−1^ at 1 A g^−1^ than the monometallic Cu-MOF (126 F g^−1^). The results were attributed to the coexistence of Ni and Cu elements with good cycling stability (80% retention over 1200 cycles). An ASC based on Ni@Cu-MOF and activated carbon (as positive and negative electrodes) delivered a specific capacitance of 48.7 F g^−1^ at 1 A g^−1^ and a high energy density of 17.3 W h kg^−1^ at a power density of 798.5 W kg^−1^. These results show that bimetallic MOFs are promising materials for SCs. This work encourages us to design and fabricate multicomponent MOF electrode materials for SCs.

## Supplementary Materials

The following are available online at https://www.mdpi.com/2073-4360/11/5/821/s1, Figure S1, TEM of Cu-MOF; Figure S2, TEM of Cu-MOF; Figure S3, Pore-size distribution of Cu-MOF; Figure S4, Pore-size distribution of Ni@Cu-MOF; Figure S5, CV curves of Cu-MOF at scan rates 2, 5, 8, 10, 20, 50, 80, 100 mV s^−1^; Figure S6, Galvanostatic charge/discharge curves of Cu-MOF at different current densities; Figure S7, CV curves of active carbon at scan rates 2, 5, 8, 10, 20, 50, 80, 100 mV s^−1^; Figure S8, Galvanostatic charge–discharge curves of active carbon at different current densities.
