# Supplementary material for: Room-Temperature Fabrication of a Nickel-Functionalized Copper Metal–Organic Framework (Ni@Cu-MOF) as a New Pseudocapacitive Material for Asymmetric Supercapacitors
Source: Polymers (Basel). 2019 May 7;11(5):821. doi: 10.3390/polym11050821 (PMC6572177; doi:10.3390/polym11050821)
Supplement: Supplementary file 1 [file polymers-11-00821-s001.zip › supporting information-polymers.pdf]

Room temperature fabrication of nickel functionalized copper metal-organic framework (Ni@Cu-MOF) nanosheets as new pseudocapacitive material for asymmetric supercapacitor

Yi Wang[a], Shengqiang Nie[a], Yuan Liu[a], Wei Yan[a], Gang cheng[a], Shaomin Lin[b], Huan Yang[b*] and Jun Luo[a*]

[a] College of Chemistry and Material Engineering, Gui Yang University, Guiyang, 550005, PR China

[b] School of Material Science and Engineering Han Shan Normal University, Chaozhou, 521041, P.R. China.

Electrochemical study in a three-electrode system.

The capacitance can be calculated according to following eqn (1): [1]

$$C = (I\Delta t)/(m\Delta V) \qquad (1)$$

where C (F g $^{-1}$) is the capacitance, ΔV is the potential range (V), I is the discharge current (A) and Δt represent the discharge time (s).

Electrochemical study in a two-electrode system.

A simple ASC was based on Ni@Cu-MOF (positive electrode) and activated carbon (negative electrode), respectively. The specific capacitance of Ni@Cu-MOF and AC are deduced from the galvanostatic discharge experiments in two-electrode system.

The weight of the active materials was determined according to the following equation: [43]

$$m^{+} / m^{-} = C^{-}V^{-} / C^{+}V^{+} \qquad (2)$$

where C, m, V are the capacitance, the mass loading, the potential window, respectively. The energy density (E, Wh/kg) and power density (P, W /kg) of the ASC were determined according to the following equations. [43]

$$E = (C\Delta V^{2})/2\times 3.6 \qquad (3)$$

$$P = 3600E/\Delta t \qquad (4)$$

where C, ΔV and Δt the specific capacitance of asymmetric supercapacitor, the potential window and the discharge time.

Preparation of electrode

The working electrodes were prepared by dispersing the active electrode material (Cu-MOF or Ni@Cu-MOF) (80%), acetylene black (10%) as conductive agent and polyvinylidene fluoride (PVDF) (10%) as a binder in N-methyl-2-pyrrolidinone (NMP). The slurry was pasted on Ni foam (1 cm ^{2} area and 1 mm thick) and dried at 60 ℃ overnight in the air. Pt plate and Hg/HgO were as the counter electrode and reference electrode, respectively.

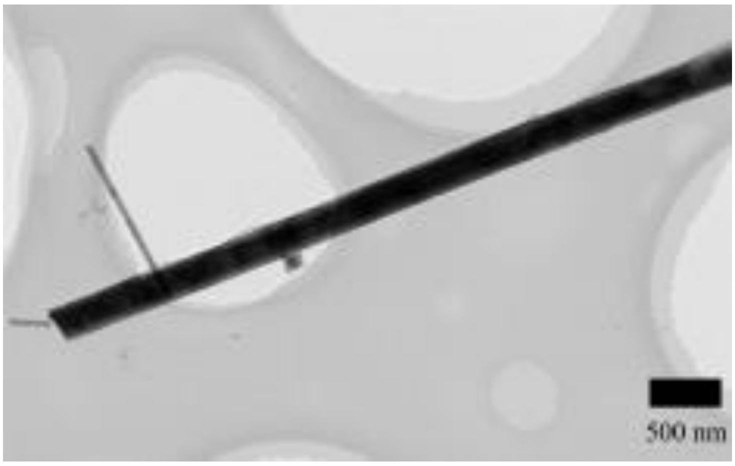

Figure S1 TEM of Cu-MOF

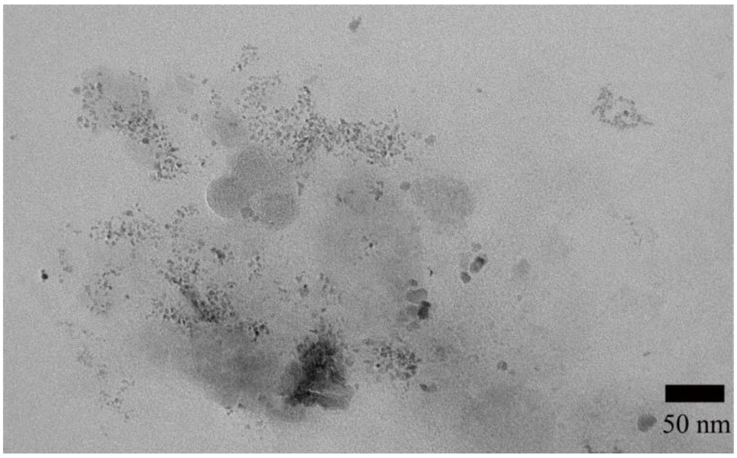

Figure S2 TEM of Cu-MOF

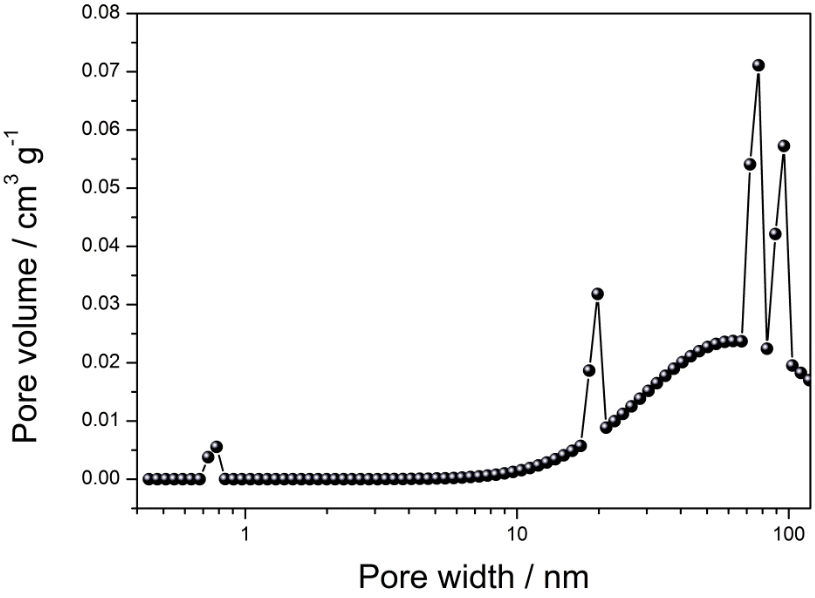

Figure S3 Pore-size distribution of Cu-MOF

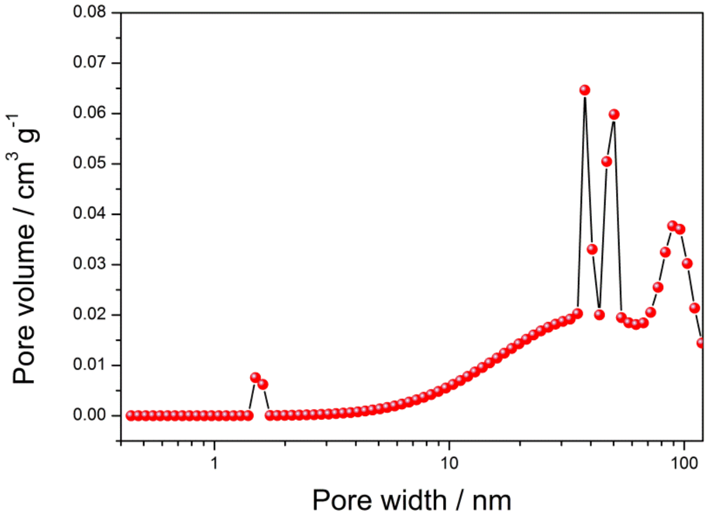

Figure S4 Pore-size distribution of Ni@Cu-MOF

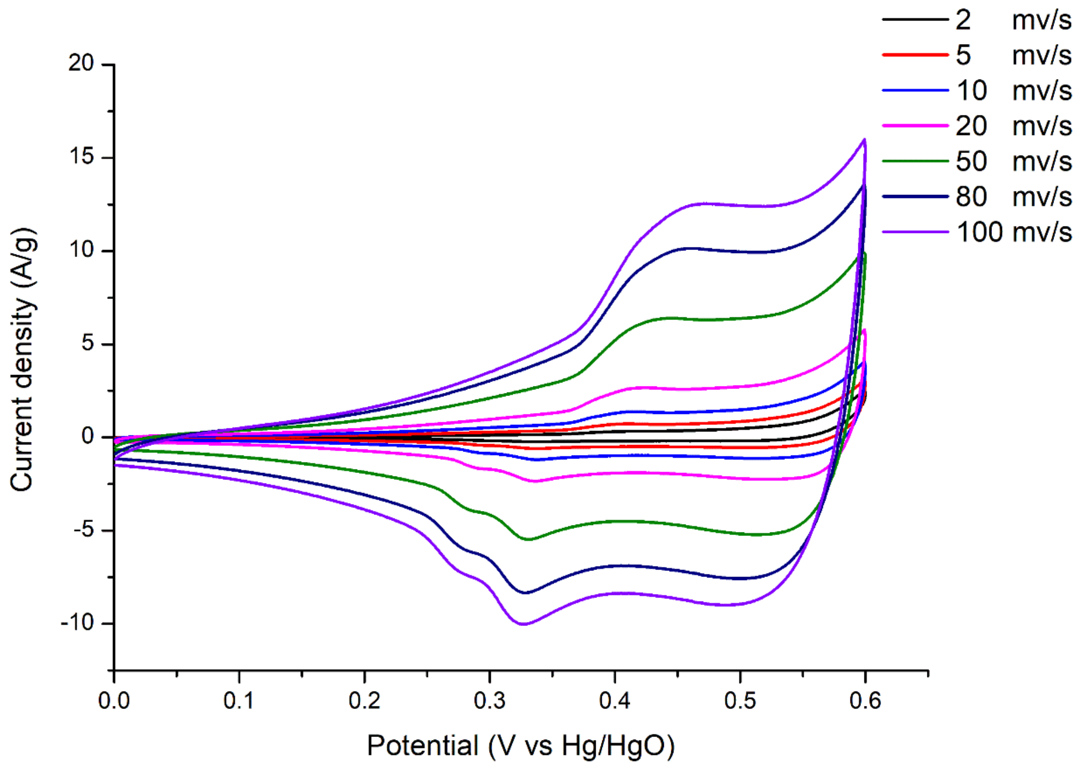

Figure S5. CV curves of Cu-MOF at scan rates 2, 5, 8, 10, 20, 50, 80, 100 mV s $^{-1}$

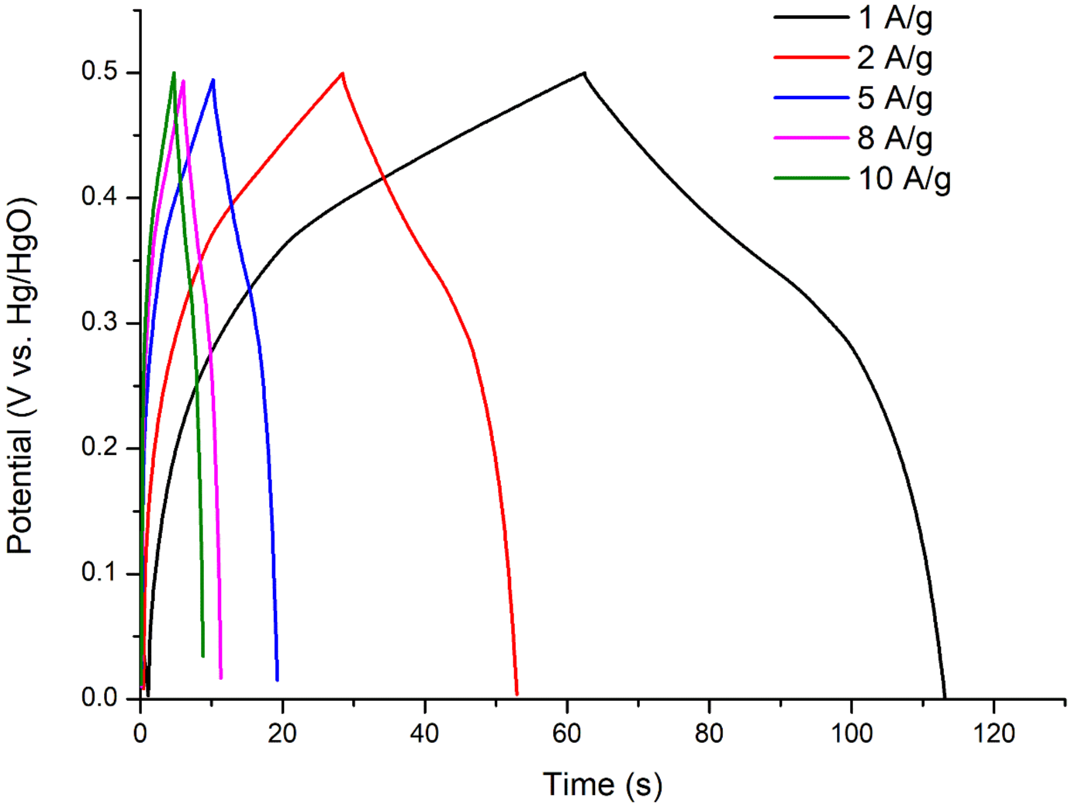

Figure S6. Galvanostatic charge/discharge curves of Cu-MOF at different current densities.

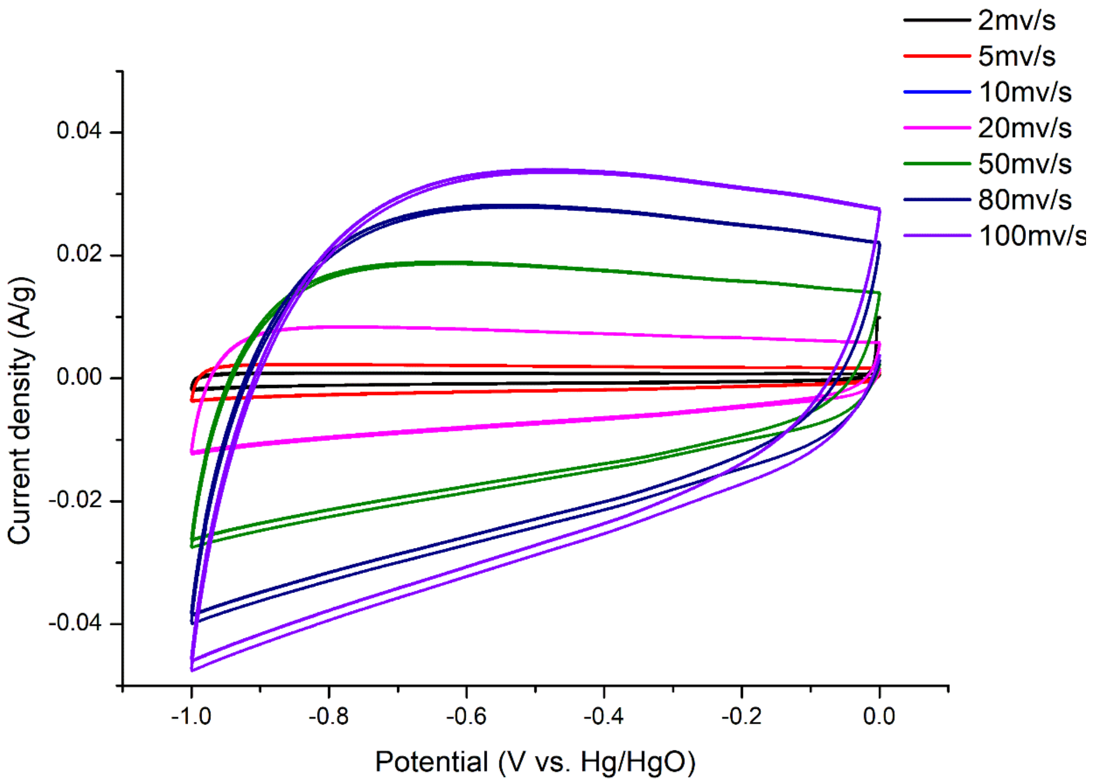

Figure S7. CV curves of actived carbon at scan rates 2, 5, 8, 10, 20, 50, 80, 100 mV s $^{-1}$

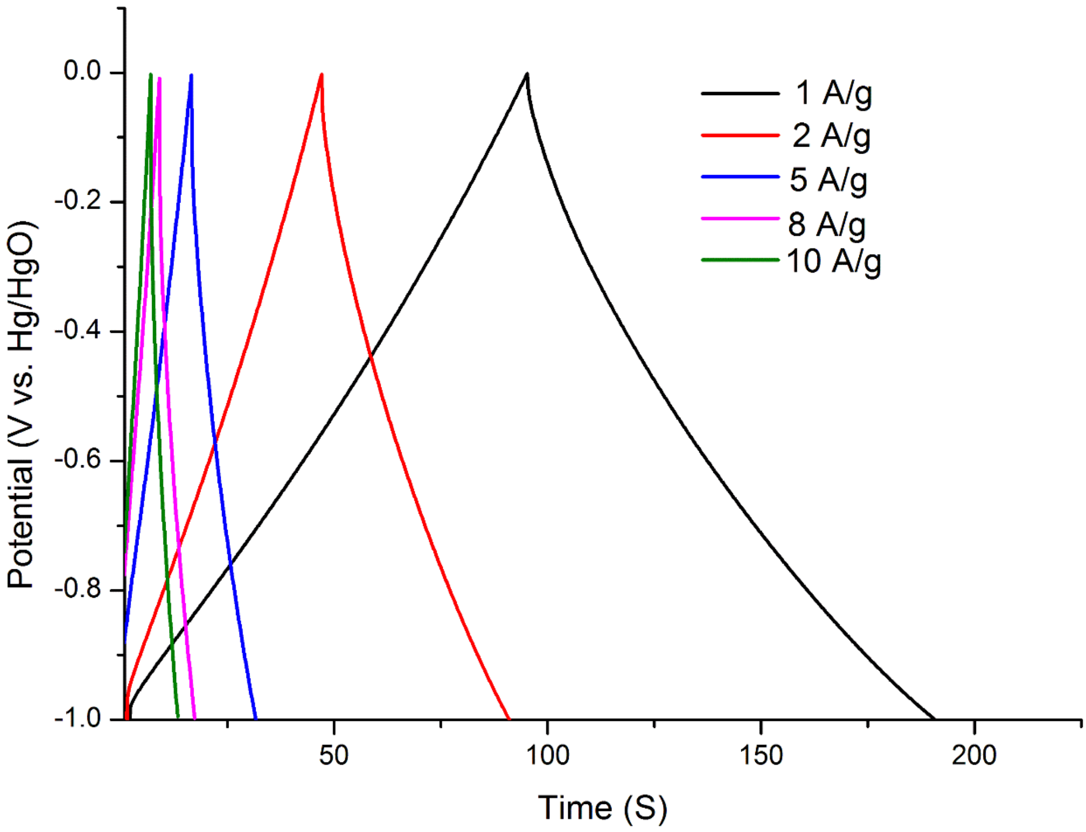

Figure S8. Galvanostatic charge/discharge curves of actived carbon at different current densities.

References

1. S. Z., Li D. H. , Chen S. , Yang X. F. , Zhao X. L. , Zhao Q. S. , Komarneni S. , Yang D. J. Highly Stable Supercapacitors with MOF-derived Co 9 S 8 /Carbon Electrodes for High Rate Electrochemical Energy Storage. J. Mater. Chem. A, 2017, 5,12453-12461.
